# The Effect of Riboflavin/UVA Collagen Cross-linking Therapy on the Structure and Hydrodynamic Behaviour of the Ungulate and Rabbit Corneal Stroma

**DOI:** 10.1371/journal.pone.0052860

**Published:** 2013-01-17

**Authors:** Sally Hayes, Christina S. Kamma-Lorger, Craig Boote, Robert D. Young, Andrew J. Quantock, Anika Rost, Yasmeen Khatib, Jonathan Harris, Naoto Yagi, Nicholas Terrill, Keith M. Meek

**Affiliations:** 1 Structural Biophysics Research Group, School of Optometry and Vision Sciences, Cardiff University, Cardiff, United Kingdom; 2 Japan Synchrotron Radiation Research Institute, Spring-8, Hyogo, Japan; 3 Diamond Light Source, Didcot, Oxfordshire, United Kingdom; Johns Hopkins University, United States of America

## Abstract

**Purpose:**

To examine the effect of riboflavin/UVA corneal crosslinking on stromal ultrastructure and hydrodynamic behaviour.

**Methods:**

One hundred and seventeen enucleated ungulate eyes (112 pig and 5 sheep) and 3 pairs of rabbit eyes, with corneal epithelium removed, were divided into four treatment groups: Group 1 (28 pig, 2 sheep and 3 rabbits) were untreated; Group 2 (24 pig) were exposed to UVA light (3.04 mW/cm^2^) for 30 minutes and Group 3 (29 pig) and Group 4 (31 pig, 3 sheep and 3 rabbits) had riboflavin eye drops applied to the corneal surface every 5 minutes for 35 minutes. Five minutes after the initial riboflavin instillation, the corneas in Group 4 experienced a 30 minute exposure to UVA light (3.04 mW/cm^2^). X-ray scattering was used to obtain measurements of collagen interfibrillar spacing, spatial order, fibril diameter, D-periodicity and intermolecular spacing throughout the whole tissue thickness and as a function of tissue depth in the treated and untreated corneas. The effect of each treatment on the hydrodynamic behaviour of the cornea (its ability to swell in saline solution) and its resistance to enzymatic digestion were assessed using in vitro laboratory techniques.

**Results:**

Corneal thickness decreased significantly following riboflavin application (p<0.01) and also to a lesser extent after UVA exposure (p<0.05). With the exception of the spatial order factor, which was higher in Group 4 than Group 1 (p<0.01), all other measured collagen parameters were unaltered by cross-linking, even within the most anterior 300 microns of the cornea. The cross-linking treatment had no effect on the hydrodynamic behaviour of the cornea but did cause a significant increase in its resistance to enzymatic digestion.

**Conclusions:**

It seems likely that cross-links formed during riboflavin/UVA therapy occur predominantly at the collagen fibril surface and in the protein network surrounding the collagen.

## Introduction

The most anterior part of the ocular system, the cornea, is tough and transparent. It offers protection to the inner contents of the eye and facilitates the passage of light onto the retina. The cornea is also a powerful refracting surface and provides the eye with up to 75% of its focussing power. The stroma, which comprises about 90% of the total thickness of the cornea, is composed mainly of water, collagen and proteoglycans. The arrangement of corneal collagen is such that, uniformly narrow fibrils lie parallel to each other in layers (lamellae) which are themselves organized in an ordered, lattice-like configuration [1], [2]. The transparency of the cornea is largely dependent on the narrow diameter and short-range order of the collagen fibrils which in turn is regulated by close interactions with proteoglycans. It is generally believed that proteoglycans act as interfibrillar spacers via the attachment of their core proteins to collagen fibrils [3] and the lateral projection of their highly sulphated glycosaminoglycan side chains which form a hydrophillic coating around the fibrils. The fibril coating is thought to counteract the attractive force caused by the thermal motion of the proteoglycan-glycosaminoglycan complex [4].

In the condition keratoconus (a leading cause of corneal transplant surgery in the United Kingdom [5]), the cornea progressively thins, weakens and assumes an increasingly abnormal curvature, thus causing a distortion of vision via incorrect focussing of light onto the retina. The cause of keratoconus and the mechanism by which it progresses are uncertain and until recently, treatments for the condition have been limited to correcting the refractive error associated with the condition and not the underlying cause. Corneal collagen cross-linking therapy is a relatively new treatment aimed at halting keratoconus progression [6]. The treatment involves the de-epithelialised cornea being pre-soaked with a photosensitiser (riboflavin/vitamin B_2_) and then exposed to ultraviolet A light (UVA). This has the effect of increasing the biomechanical stability of the cornea [7], [8] and its resistance to enzymatic digestion [9], thereby arresting keratoconus progression. The potential of the technique for the treatment of other corneal disorders such as keratitis [10], [11] and bullous keratopathy [12], [13], [14], [15], [16] is currently being explored.

To date, little is known about the specific nature of the cross-links that are formed as a result of riboflavin/UVA collagen cross-linking, although it has been suggested that the effect of the treatment is carbonyl dependent and involves the formation of advanced glycation end product cross-links [17]. There is also sparse knowledge about the precise location of the cross-links formed, either within collagen fibrils or in the interfibrillar matrix, however, the absence of any change in the cohesive strength of the cornea following treatment suggests that cross-links are not formed between collagen lamellae [18]. In principle, many of the results obtained to date could be explained by assuming cross-links occur within or between collagen molecules, between the surfaces of the collagen fibrils or between the fibrils and the proteoglycan-rich matrix. Zhang et al. [19], using an *in vitro* model reaction system consisting of purified proteins in solution, have shown that UVA irradiation in the presence of riboflavin results in both collagen-collagen and proteoglycan-proteoglycan intermolecular cross-linking and limited linkages between collagen molecules and proteoglycan core proteins. The formation of cross-links between the major interstitial collagens and other stromal collagens and/or proteoglycans would be expected to influence the hydrodynamic behaviour of the cornea. However, despite Wollensak et al. [20] showing that riboflavin/UVA cross-linked corneas swell less than untreated corneas, it remains unclear from their study whether the observed resistance to stromal swelling was due to collagen cross-linkage or the presence of riboflavin solution (containing the deturgescent agent dextran) within the treated tissue.

Here we use high and low-angle x-ray scattering, laboratory swelling techniques and enzymatic digestion to conduct a detailed study of corneal ultrastructure, hydrodynamic behaviour and enzymatic resistance following riboflavin/UVA cross-linkage therapy. The aim of the study is to improve understanding of the technique and provide a sound scientific basis for its use in the treatment of other corneal disorders. X-ray scattering is non-invasive and provides information about the separation of molecules within the collagen fibrils and about the distance by which they are separated from each other [21]. It also yields details about the axial structure of the collagen fibrils and about the lateral organisation of the packing of collagen fibrils, a parameter of some importance for corneal transparency [21]. The data obtained are averages from every collagen molecule or fibril in the thickness of the cornea through which the x-rays pass, and are thus highly representative of the tissue as a whole.

## Methods

### Tissue

A total of one hundred and seventeen ungulate eyes (112 pig and 5 sheep) with no visible signs of corneal scarring or opacity were obtained within 24 hours of death from a local EC licensed abattoir (Ensors Gloucestershire Ltd., UK). The abattoir granted permission for the eyes to be used in research. A further 3 pairs of rabbit eyes (obtained within 29 hrs of death) were purchased from a licensed commercial food source producing Human Grade Consumption Meats (Woldsway Foods Ltd., UK); full permission was given for their use in research. In the case of corneas prepared for enzymatic digestion studies and x-ray scattering investigations, the epithelium was carefully removed using a razor blade and one of four treatments (described below) was applied to the anterior stromal surface. In order to study the effect of cross-linking on the hydrodynamic behaviour of the corneal stroma it was necessary to remove both the epithelium and endothelium and apply treatment to the posterior stroma, since corneal swelling is known to occur mainly in the posterior stroma [22], [23], [24].

The treatment groups were:

**Group 1**: Untreated controls (pig, n = 28; sheep, n = 2; rabbit, n = 3).

Eyes in this group were left uncovered on the laboratory bench (with the cornea facing upwards) for 30 minutes.

**Group 2**: UVA only (pig, n = 24):

The cornea was exposed to UVA irradiation (3.04 mW/cm^2^) for 30 minutes using a commercial UVA illumination system (UV-X™, IROC, Switzerland) with a surface irradiance of 3.04 mW/cm^2^, a focussing distance of 5 cm and an illumination diameter of 9 mm.

**Group 3**: Riboflavin only (pig, n = 29).

Riboflavin eye drops (riboflavin 0.136% and dextran T500 25%) were applied to the surface of the cornea at 5 minute intervals for 35 minutes. During the treatment, the cornea was covered by a black box to prevent any interaction with light.

**Group 4**: Riboflavin/UVA cross-linked (pig, n = 31; sheep, n = 3; rabbit, n = 3).

Riboflavin eye drops (riboflavin 0.136% and dextran T500 25%) were applied to the surface of the cornea at 5 minute intervals for 35 minutes. Five minutes after the initial riboflavin application the corneas were exposed to UVA light (3.04 mW/cm^2^) for 30 minutes whilst the application of riboflavin eye drops continued.

After treatment, each cornea (with a 2 mm scleral rim) was wrapped in Clingfilm™ (Superdrug Stores Plc., Croydon, UK) to prevent moisture loss. Corneas destined for x-ray scattering studies (pig = 64; sheep = 5; rabbit = 6) were frozen at −80°C until required for data collection, whilst the 36 pig corneas prepared for swelling studies were chilled overnight at 4°C. The twelve corneas prepared for enzymatic digestion were used immediately.

### Enzymatic Digestion Studies

An 8.25 mm corneal button was trephined from the centre of twelve pig corneas (3 from each group). The corneal disks were then immersed into individual plastic tubes containing 8 ml of pepsin solution (1 g purified pepsin (Sigma, Munich) in 10 ml 0.1 M HCl at pH 2.2). The diameter of the corneal disks was recorded daily for eleven days.

### X-ray Scattering Studies

Thirty minutes prior to x-ray data collection, each cornea (pig = 64; sheep = 5; rabbit = 6) was thawed at room temperature; it has been previously shown that the intermolecular and interfibrillar spacing of corneal collagen returns to physiological levels upon thawing [25]. Immediately prior to data collection, an 8.25 mm button was trephined from the centre of 12 pig corneas in Group 1, 12 pig corneas in Groups 2, 17 pig corneas in Group 3 and 17 pig corneas in Group 4. In another 6 pig and 5 sheep corneas (Groups 1 = 3 pig and 2 sheep; Group 4 = 3 pig and 3 sheep), a thin strip of tissue, measuring approximately 0.6 mm wide, was dissected from the centre of each cornea in the superior/inferior direction. After dissection, each corneal button/strip was re-wrapped in Clingfilm to prevent tissue dehydration during data collection.

### Small-angle X-ray Scattering Data Collection and Analysis

Small-angle x-ray scattering patterns were obtained from the centre of 32 pig corneal buttons (8 corneas from each group) on Station 2.1 at the original UK Synchrotron Radiation Source (Daresbury, UK). Each pattern was generated from a 60–90 second exposure to a 1×1 mm x-ray beam with a wavelength of 0.1544 nm and recorded on a detector positioned 7.5–7.75 m behind the sample.

Owing to the sensitivity of collagen interfibrillar spacing to changes in corneal hydration [26] it was necessary to calculate the hydration of each corneal button at the time of data collection. This was done using Equation 1.
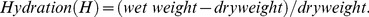
(1)


Equivalent small-angle x-ray scatter patterns were collected from the centre of 6 rabbit corneas (Groups 1 = 3; Group 4 = 3) on Beamline I22 at the new UK synchrotron radiation source (Diamond Light Source, Didcot, UK) using a 150 s exposure to a 0.25×0.25 mm x-ray beam (λ = 0.1 nm). The x-ray scatter patterns were collected on a detector positioned 6.2 m behind the sample. The thickness of each cornea was measured before and after data collection using a Pachette2™ Ultrasonic Pachymeter (DGH Technology, USA).

**Figure 1:**
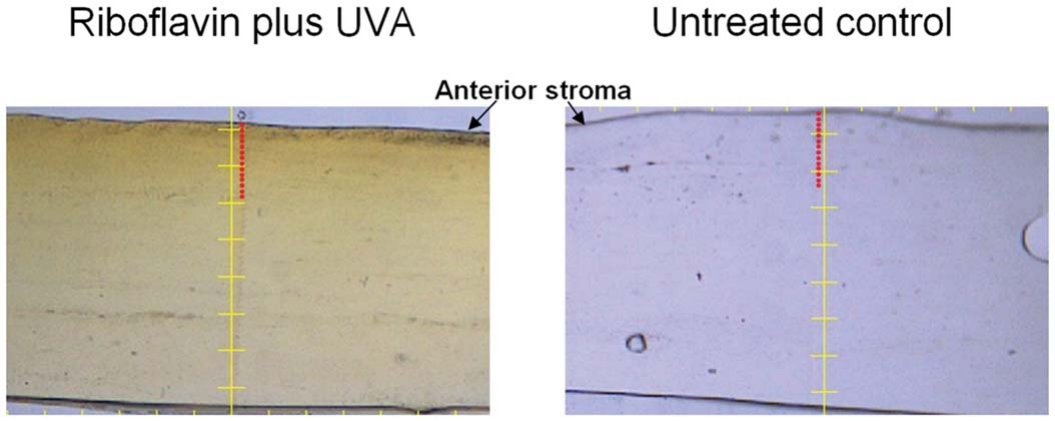
Cross-section of cross-linked and non-cross-linked pig corneas. Small-angle x-ray scattering images were obtained at 25 µm intervals (red circles) throughout the anterior 300 µm of riboflavin/UVA treated and untreated strips of pig cornea using a microfocus x-ray beam. Each graduation on the scale bar represents 150 µm.

On beamline 40 XU at the SPring-8 synchrotron radiation source (Hyogo, Japan), strips of pig corneas from Groups 1 (n = 3) and 4 (n = 3) were positioned so that their cut edge was perpendicular to the incident beam direction. Small-angle x-ray scatter patterns were obtained at 25 µm intervals throughout the anterior 300 µm of the tissue using a 0.015 s exposure to a circular microfocus x-ray beam (λ = 0.118 nm) measuring 25 µm in diameter at the specimen (Figure 1). The patterns were recorded on a detector placed 3 m behind the specimen. X-ray scattering data collection was limited to the anterior 300 µm of the cornea for two reasons: firstly, the cross-linking treatment is maximal in the anterior 300 µm of the cornea [27], [28] and secondly, changes in corneal hydration (which affect collagen interfibrillar spacing [26] and is caused in this case by the application of the deturgescent agent dextran to the cross-linked corneas), occur predominantly in the posterior stroma [29].

All small-angle x-ray scatter patterns were calibrated against the 67 nm *meridional* spacing (D-periodicity) of collagen in hydrated rat tail tendon. The collagen axial D-periodicity in the treated and untreated corneas was then evaluated from the *meridional* diffraction reflections and the modal average inter-fibrillar Bragg spacing of corneal collagen fibrils was determined from the position of the interfibrillar *equatorial* reflection [21], [30]. The degree of local ordering of the collagen fibrils was expressed as a *spatial order factor,* which can be calculated by measuring the ratio of the height of the interfibrillar diffraction peak to the width at half height [31]. The absolute number of this ratio is arbitrary, but the larger the number, the higher the degree of order in the packing arrangement.

### Wide-angle X-ray Scattering Data Collection and Analysis

Wide-angle scattering patterns were obtained from the centre of 26 pig corneal buttons (Group 1 = 4; Group 2 = 4; Group 3 = 9; Group 4 = 9) using a 10 second exposure to a 0.2×0.2 mm focussed x-ray beam with a wavelength of 0.1488 nm on Station 14.1 at the Daresbury synchrotron radiation source. The patterns were recorded on a detector placed 150 mm behind the sample. In order to calculate the modal average inter-molecular spacing of corneal collagen the system was calibrated using the 0.305 nm lattice reflection in powder diffraction patterns of calcite.

A microfocus x-ray beam (λ = 0.13 nm) of dimensions 300×50 µm (horizontally×vertically) on SWING station at Soleil synchrotron radiation source (Paris, France) was used to obtain wide-angle x-ray scatter patterns at 50 µm intervals throughout the anterior 300 µm of 5 sheep corneal strips (Group 1 = 2; Group 4 = 3) mounted edge-on in the direction of the x-ray beam. Each pattern, resulting from a 20 s exposure to the x-ray beam, was recorded on a detector located 520 mm behind the specimen and calibrated against the 5.8 nm lattice reflection from silver behenate.

The relationship between x-ray Bragg spacing and the corresponding centre-to-centre distance of the parameter under investigation depends on the precise packing of the molecules within the fibrils, or of the fibrils within the stroma. Most previous investigations have assumed a liquid-like packing [32], [33], in which case Bragg spacings need to be multiplied by a factor of 1.1–1.2 in order to convert to centre-to-centre spacings. However, as we are only concerned here with changes in these parameters, we present all results as Bragg spacings.

### In vitro Stromal Swelling Studies

Twenty-four pig corneas (Group 1 = 7; Group 2 = 6; Group 3 = 6; Group 4 = 5) were used to examine the hydrodynamic behaviour of the stroma immediately after treatment. Before treatment, all swollen corneas were air dried until they reached a near physiological de-epithelialised thickness of 651±26 µm (measured using an ultrasound pachymeter) [34]. Following treatment, the corneas in Groups 1 and 2 were air dried until they reached a similar thickness to that of the corneas in Groups 3 and 4 (548±34 µm). A 7.25 mm corneal disk was then trephined from the centre of each cornea and the weight of the disk recorded (Time 0). The corneal disks were then placed into individual tubes containing 3 ml of 0.9% saline solution and allowed to swell freely. During the first hour the saline solution was changed every 15 min (to avoid a build-up of dextran in the bathing medium of Groups 3 and 4). The wet weight of each specimen (after removal of excess fluid) was recorded at regular 15 min intervals for 5 hrs. Upon completion the corneal disks were oven dried for 3 days and the hydration at each time point calculated using Equation 1.

A further 12 pig corneas (Group 1 = 3; Group 2 = 3; Group 3 = 3; Group 4 = 3) were used to study the longer term swelling behaviour of treated and untreated corneas and establish the maximum achievable hydration for each treatment group. Immediately after their respective treatments, a 7.25 mm corneal disk was trephined from the centre of each cornea and its weight recorded (Time 0). The corneal disks were then placed in individual tubes containing 3 ml of 0.9% saline solution with 3 mM sodium azide (to prevent bacterial growth) and allowed to swell freely. At regular intervals the wet weight of each specimen was recorded and the saline solution was changed. After twenty-three days of swelling the corneal disks had reached a stable wet weight and the study was terminated. The samples were oven dried for three days and the hydration at each measured time point was calculated using Equation 1.

### Statistical Analyses

In most cases, differences between treatment groups and their controls were statistically evaluated using ANOVA. In cases where the F-test p-value was less than p<0.05, the null hypothesis was rejected and a pair-wise comparisons of means was performed using the least significant difference method. For paired samples (rabbit corneas) statistical differences between treatment groups were assessed by means of a paired student t-test. A difference between any pair of means of greater than or equal to the least significant difference at p<0.05 was considered to be statistically significant. Values presented in the results are means ± standard deviation (SD).

## Results

### Enzymatic Digestion

**Figure 2:**
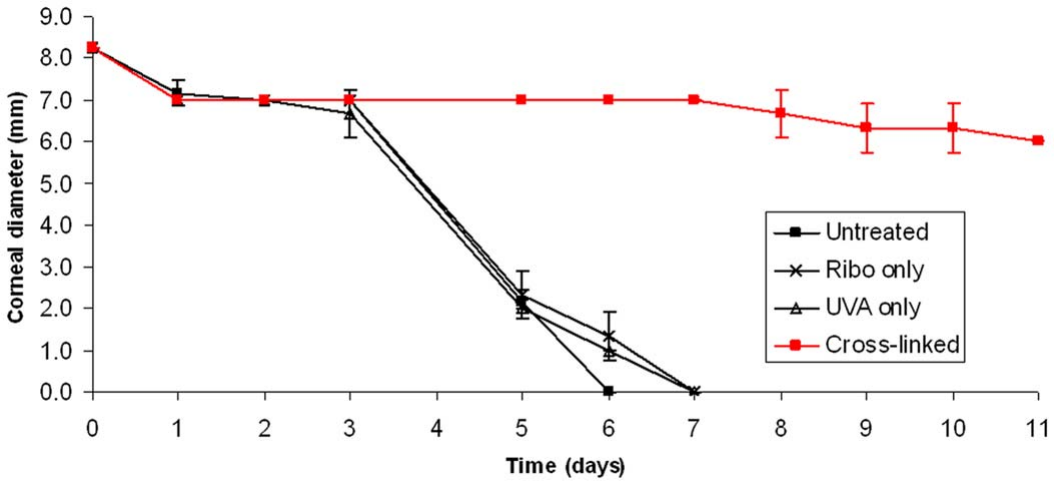
Enzymatic digestion rate of treated and untreated pig corneas.

After six days in pepsin solution all non-cross-linked corneas (Groups 1, 2 and 3) were completely digested (Figure 2). At the same time-point, the average diameter of the riboflavin/UVA cross-linked specimens had decreased by only 21% and the anterior curvature of the cornea remained visible.

### Collagen Interfibrillar Spacing, Fibril Diameter, D-periodicity and Spatial Order

Using calibrated small-angle x-ray scatter patterns from treated and untreated pig and rabbit corneas, the average interfibrillar Bragg spacing, spatial order factor, diameter and D-periodicity of the collagen fibrils were calculated (Tables 1 and 2).

**Table 1:** Average collagen interfibrillar spacing, D-periodicity and order factor in treated and untreated pig corneas.

	Group 1 (n = 8)(Untreated)	Group 2 (n = 8)(UVA only)	Group 3 (n = 8)(Riboflavin only)	Group 4 (n = 8)(UVA+riboflavin)
Hydration	5.3 (+/−0.8)	5.2 (+/−0.7)	4.6 (+/−0.5)	4.6 (+/−0.4)
IFS* (nm)	61.7 (+/−2.1)	61.8 (+/−0.9)	60.6 (+/−1.6)	60.3 (+/−1.6)
Spatial order factor	**23.9 (+/−3.2)**	25.2 (+/−6.0)	27.9 (+/−5.4)	**33.6 (+/−3.6)**
Fibril diameter	35.7 (+/−0.6)	36.0 (+/−0.5)	36.2 (+/−0.6)	35.8 (+/−0.4)
D-period (nm)	65.0 (+/−0.2)	65.0 (+/−0.2)	65.0 (+/−0.3)	64.9 (+/−0.3)

* IFS = Interfibrillar Bragg spacing.

Significant differences between treatment groups of p<0.01 are highlighted in bold type.

**Table 2:** Average collagen interfibrillar spacing, D-periodicity and order factor in treated and untreated paired rabbit corneas.

	Group 1 (n = 3)(Untreated)	Group 4 (n = 3)(UVA+riboflavin)
Thickness	384 (+/−8.7)	405.3 (+/−27.2)
IFS* (nm)	47.7 (+/−4.0)	53.3 (+/−1.8)
Spatial order factor	44.3 (+/−8.2)	55.4 (+/−10.7)
Fibril diameter	38.4 (+/−1.7)	40.3 (+/−0.5)
D-period (nm)	66.1 (+/−0.2)	66.0 (+/−0.0)

* IFS = Interfibrillar Bragg spacing.

A 30-minute exposure to UVA light did not produce any significant change in the average interfibrillar Bragg spacing, spatial order, fibril diameter or D-periodicity in either riboflavin-treated (Group 4) or non-riboflavin treated (Group 2) pig corneas (Table 1). However, when compared to untreated pig corneas (Group 1) the fibril spatial order factor was significantly higher in the cross-linked samples (Group 4) (P<0.01).

A paired t-test revealed no significant difference in the average interfibrillar Bragg spacing, spatial order factor, diameter or D-periodicity of stromal collagen between the riboflavin/UVA treated rabbit corneas and their untreated pairs (Table 2).

**Figure 3:**
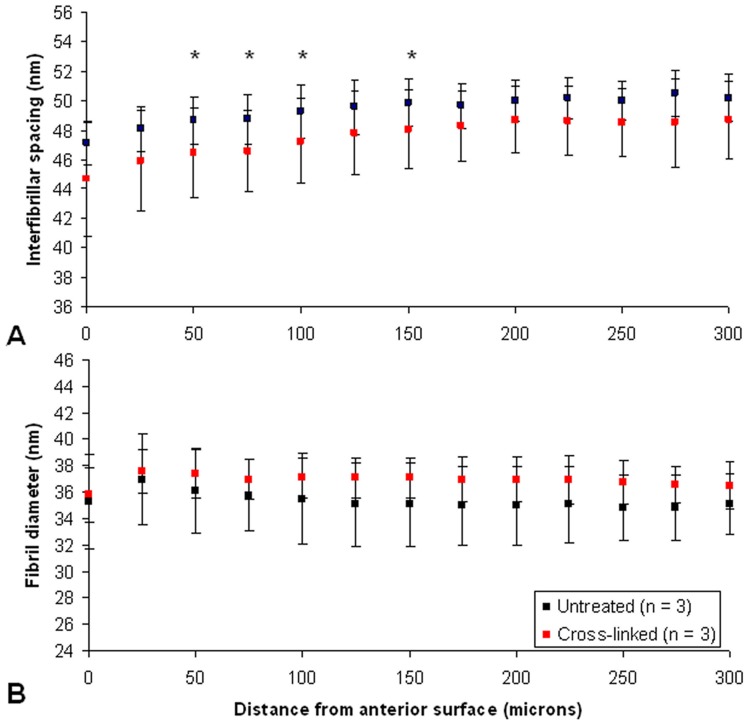
Collagen parameters in cross-linked and non-cross-linked pig corneas. Average measurements of interfibrillar spacing (A) and fibril diameter (B) at 25 µm intervals throughout the anterior 300 µm of treated and untreated pig corneas. Significant differences between treatment groups are highlighted by asterices (p<0.05).

Microfocus small-angle x-ray scattering data obtained from strips of riboflavin/UVA treated (Group 4) and untreated (Group 1) sheep corneas were analysed to produce through-thickness measurements of Bragg interfibrillar spacing and fibril diameter in the anterior 300 µm of the corneal stroma. Throughout most of the anterior stroma no difference in the average Bragg interfibrillar spacing was detected between the treated and untreated corneas. However, a significantly lower collagen interfibrillar spacing was observed in the less hydrated, treated corneas at 4 out of the 13 sites examined (tissue depths of 50 µm (p<0.05), 75 µm (p<0.01), 100 µm (p<0.05) and 150 µm (p<0.05) from the anterior surface) (Figure 3A).

No significant difference in fibril diameter was detected between the treated and untreated corneas within the anterior 300 µm of the tissue (Figure 3B).

### Collagen Intermolecular Spacing

**Figure 4:**
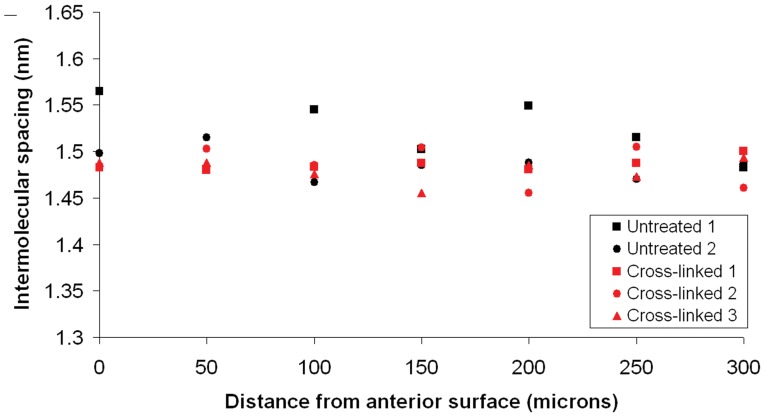
Depth profile of collagen intermolecular spacing in treated and untreated sheep corneas.

The intermolecular Bragg spacing of collagen was calculated for both treated and untreated pig corneas using calibrated wide-angle x-ray scatter patterns (Table 3). The intermolecular spacing of corneal collagen appeared to be unaffected by a 30-minute exposure to UVA light in riboflavin-treated (Group 4) and non-riboflavin treated pig corneas (Group 2). This was confirmed in a depth-dependent study of untreated (Group 1) and riboflavin/UVA treated (Group 4) sheep corneas which revealed no significant difference in collagen intermolecular spacing between the two groups in 6 out of the 7 measured sites located within the anterior 300 µm of the tissue (Figure 4). The only exception to this occurred at a distance of 200 µm from the anterior surface; at this location the average intermolecular spacing of the untreated corneas was found to be higher than that of the less hydrated cross-linked corneas (p<0.05).

**Table 3:** Average collagen intermolecular spacing in treated and untreated pig corneas.

	Group 1 (n = 4)(Untreated)	Group 2 (n = 4)(UVA only)	Group 3 (n = 9)(Riboflavin only)	Group 4 (n = 9)(UVA+riboflavin)
Hydration	4.4 (+/−0.6)	4.7 (+/−0.3)	4.4 (+/−0.5)	4.2 (+/−0.3)
IMS* (nm)	1.56 (+/−0.01)	1.56 (+/−0.02)	1.57 (+/−0.02)	1.57 (+/−0.02)

* IMS = intermolecular Bragg spacing shown in A. Standard deviation is shown in brackets.

### Hydrodynamic Behaviour of the Cornea

**Figure 5:**
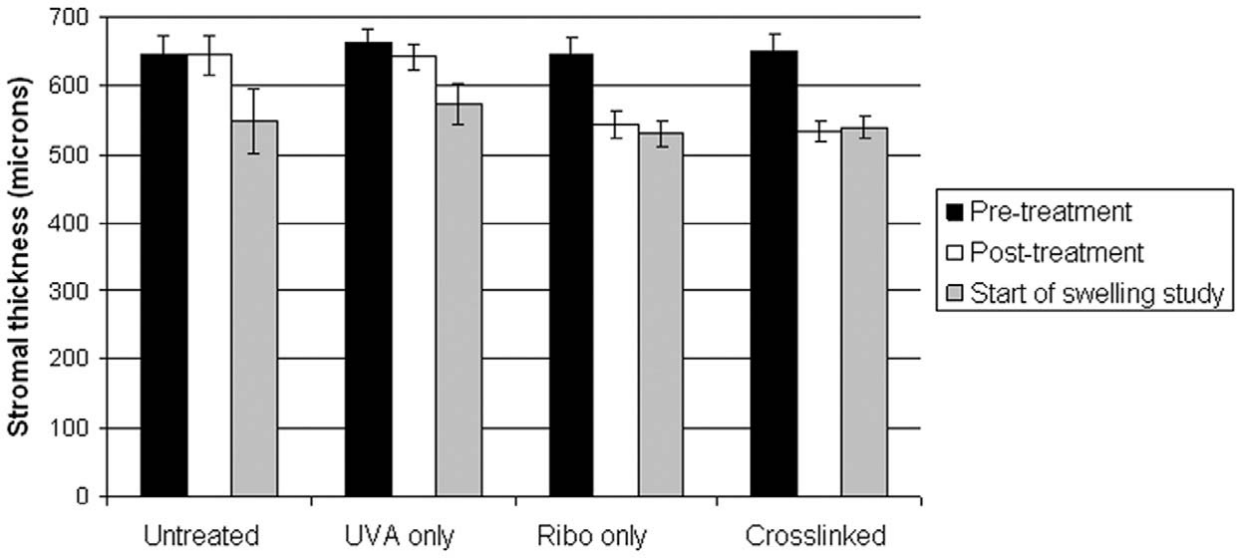
Stromal thickness in pig corneas before and after treatment. Controlled air drying of the most hydrated corneas ensured that all corneas were of a similar thickness at the start of the swelling study.

A significant reduction in stromal thickness was observed following riboflavin application in both the irradiated and non-irradiated groups (P<0.01) (Figure 5). Stromal thickness also decreased, albeit to a lesser extent in corneas exposed to UVA in the absence of riboflavin (p<0.05). By air drying the most swollen corneas we ensured that there was no significant difference in stromal thickness between treatment groups at the start of the swelling study (Figure 5).

**Figure 6:**
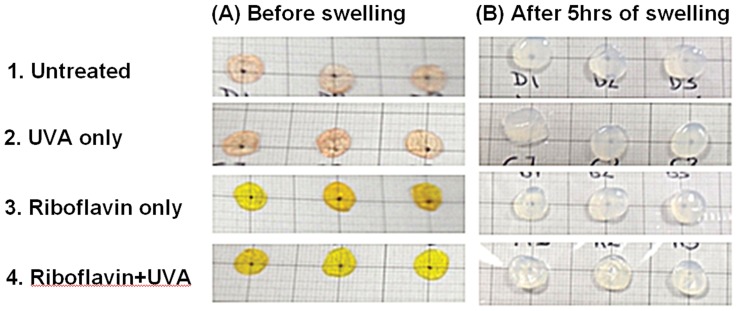
Treated and untreated corneal buttons before and after in vitro swelling. A black dot was drawn on the graph paper beneath each cornea for qualitative assessment of corneal transparency before (A) and after (B) 5 hours of swelling in saline solution.

After treatment, corneas in groups 3 and 4 had a yellow tint as a result of riboflavin penetration but all corneas remained transparent (Figure 6A). After 1 hour of swelling (and three changes of the swelling medium), the yellow colouring had disappeared from both the corneas and their swelling solution. This loss of colour was seen to indicate a removal of the riboflavin and presumably also the deturgescent dextran from within the corneal stroma, thereby allowing the ‘true’ effect of the crosslinking procedure on corneal hydrodynamic behaviour to be established. After 5 hours in the swelling medium all corneal buttons appeared opaque (Figure 6B).

**Figure 7:**
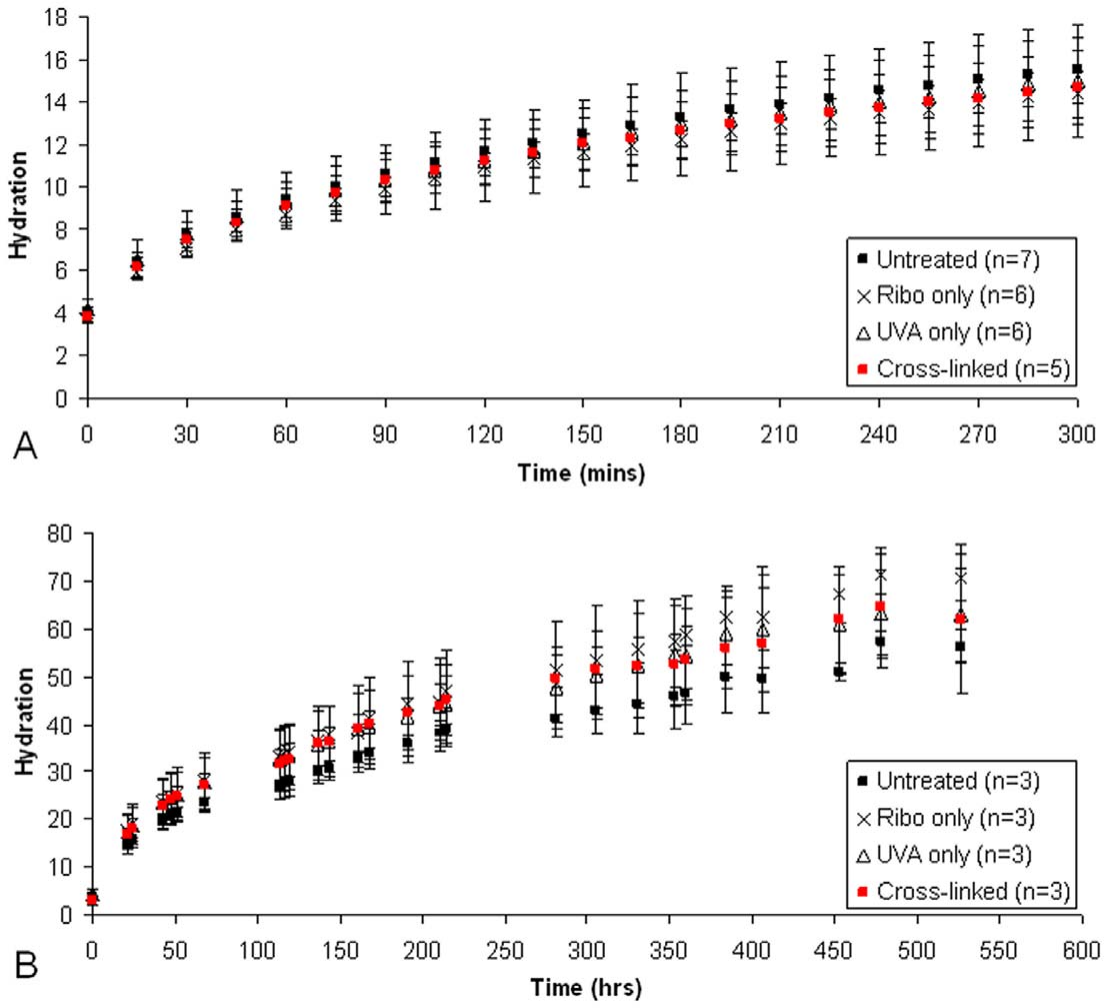
Stromal swelling rate in treated and untreated pig corneas. Data shows the rapid stromal swelling phase (A) and the subsequent slower swelling period (B).

The rate of stromal swelling did not differ between treated and untreated corneas; this was found to be the case during the initial rapid swelling phase (which was monitored closely over 5 hours) (Figure 7A) and also the subsequent period of slower stromal swelling, which stabilised after twenty-three days (Figure 7B). Measurements of final tissue hydration revealed no significant difference between treatment groups in terms of the maximum achievable stromal hydration (Table 4).

**Table 4:** Maximum achievable hydration of treated and untreated pig corneas.

	Group 1(Untreated)	Group 2(UVA only)	Group 3(Riboflavin only)	Group 4(UVA+ riboflavin)
Cornea 1	54.7	56.6	65.8	79.9
Cornea 2	60.3	73.9	75.5	50.7
Cornea 3	54.1	58.2	70.9	55.5
Average (+/−S.D)	56.4 (+/−3.4)	62.9 (+/−9.6)	70.7 (+/−4.9)	62.0 (+/−15.7)

## Discussion

The success of riboflavin/UVA cross-linking therapy for the prevention of keratoconus progression has been well documented in recent years [35], [36], however the use of the technique for the treatment of bullous keratopathy (a condition in which the cornea becomes permanently swollen), has failed to produce lasting benefits [14], [15], [16]. In order to form a better understanding of the functionality of riboflavin/UVA cross-linking for the treatment of various corneal disorders and to determine the site at which riboflavin/UVA induced cross-links are formed, we investigated the effects of the treatment on corneal ultrastructure, hydrodynamic behaviour and resistance to enzymatic digestion, The results presented in this study provide the first scientific evidence that may help to explain why riboflavin/UVA cross-linking of bullous keratopathy corneas produces only short-term improvements in corneal thickness and transparency.

Cross-linking of collagen molecules occurs as a function of age, and can be induced by incubation with sugars, or can be achieved using cross-linking reagents. It has been previously shown using x-ray scattering techniques that as the cornea ages, crosslinking leads to a 14% increase in the cross-sectional area associated with each molecule within a fibril [37] and *in vitro*, the formation of advanced glycation end-products can cause increases of up to 50% [38], [39]. Glutaraldehyde cross-linking of the cornea has been shown to increase the area associated with each corneal collagen molecule by some 11% [40]. This property, then, is a useful indicator of the occurrence of collagen cross-linking in a tissue at the molecular level.

Regrettably, in an earlier preliminary abstract [41] we reported observations of increased inter-molecular spacing following riboflavin/UVA cross-linking of pig corneas which further investigations revealed to be an artefact. In this extensive study we observed a notable absence of any change in the average intermolecular spacing of corneal collagen following UVA/riboflavin treatment. However, as the x-ray scatter pattern represents an average of every collagen molecule in the path of the beam, and riboflavin/UVA cross-linking only occurs in the most anterior 300 microns of the tissue [27], [28], it was necessary to confirm these findings by examining collagen intermolecular spacing at fine intervals throughout the thickness of the anterior cornea using a microfocus x-ray beam. Again, no major differences in collagen intermolecular spacing were detected between treated and untreated samples. Furthermore, unpublished x-ray scattering data (collected on beamline ID13 at the European Synchrotron Radiation Source, Grenoble, FR) have shown that when riboflavin/UVA cross-linked corneas are dried, collagen intermolecular spacing decreases at a normal rate, thereby demonstrating that the cross-links formed during riboflavin/UVA cross-linking are not capable of preventing molecular collapse. In addition to this, and consistent with our previous work on normal and keratoconus human corneas [42], small-angle x-ray scattering data from ungulate and rabbit corneas revealed no treatment-induced change in the collagen D-period. This result supports our belief that the riboflavin/UVA cross-linking does not have any effect on the axial stagger or the tilt of the collagen molecules within the fibrils [42], which is not wholly surprising since it is known that cross-linking corneal collagen with a strong fixative such as glutaraldehyde produces only a 0.8% reduction in the collagen D-period [40].

**Figure 8:**
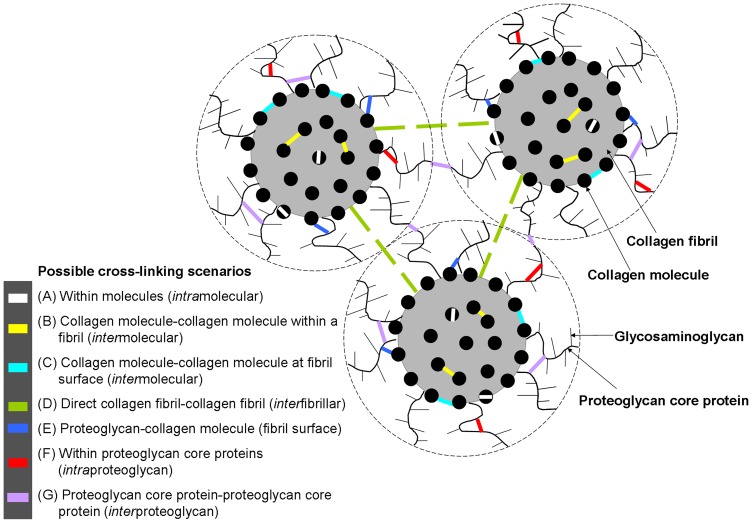
Schematic showing possible cross-linking scenarios. A simplified model showing three collagen fibrils, each with a coating (outer limit shown as a broken line) consisting mainly of proteoglycans which are attached to the fibril and form a porous network with fractal dimension (based on Fratzl and Daxer's theoretical model of collagen fibrils in the corneal stroma [48]). Coloured lines indicate the possible location(s) of riboflavin/UVA induced cross-links that are discussed in the text.

Our inability to detect molecular changes after crosslinking leads us to conclude that riboflavin/UVA therapy does not result in the wide-spread cross-linking of collagen molecules (Figure 8, scenarios A and B). However, we cannot discount the possibility of cross-link formation occurring within and/or between collagen molecules at the *surface* of collagen fibrils (Figure 8, A and C)), which may cause changes that are obscured by the averaging mechanism of the x-ray scatter technique. These surface crosslinks, once formed, would be expected to hinder cross-linking of collagen molecules located within the fibril. Such a scenario would explain the enhanced biomechanical properties of the cross-linked cornea [7], [8], the reported [9] and here confirmed increased resistance of corneal collagen to enzymatic digestion (which possibly occurs by means of steric hindrance of the cleavable sites), and the absence of any change in the *modal average* intermolecular spacing of the corneal collagen as measured by x-ray scattering.

In our human studies, the decrease in collagen interfibrillar spacing following standard riboflavin/UVA treatment and the increase in interfibrillar spacing following hypo-osmolar riboflavin cross-linking were attributed to changes in tissue hydration and did not support the existence of direct interfibrillar cross-linking (Figure 8, scenario D) [42]. Besides, in theory, direct interfibrillar cross-links are unlikely as collagen fibril surfaces are too widely separated. Although no significant changes in interfibrillar spacing were observed here following treatment, a significant increase in the spatial order factor, similar to that seen in our human study [42], was observed in the riboflavin/UVA cross-linked pig corneas. We hypothesise that the increase in short range order of collagen fibrils following riboflavin/UVA treatment may occur as a result of cross-links being formed within the fibril coating (within and between proteoglycan core proteins (Figure 8F and G)) which make the stromal collagen more resistant to the thermal motion of the proteoglycan-glycosaminoglycan complex [4]. This may also explain the reported increase in transparency of hen corneas following crosslinking [43].

Despite our x-ray scattering studies providing no evidence of direct inter-fibrillar cross-link formation (Figure 8D) following riboflavin/UVA treatment, we further investigated the possibility by searching for changes in the hydrodynamic behaviour of cross-linked corneas. This was done on the basis that when the normal cornea swells, a small amount of fluid enters the fibrils but the majority of the swelling occurs between the fibrils [42]; the presence of direct interfibrillar cross-links would therefore be expected to restrict the ability of the cornea to swell. Cross-links formed between proteoglycan core proteins and collagen molecules (Figure 8E) would also be expected to alter the hydrodynamic behaviour of the cornea, by preventing some proteoglycans from leaching out of the corneal stroma during *in vitro* swelling [44]. Although Wollensak et al. [20] have previously reported that cross-linked corneas swell less than normal their work was carried out by crosslinking the anterior cornea, which does not swell to any great extent and the riboflavin solution containing the deturgescent agent, dextran, was only applied to the UVA-treated corneas and not to their controls. It was therefore unclear from this study whether the observed resistance to stromal swelling in the cross-linked corneas was due to presence of dextran within the tissue or to inter-fibrillar crosslinking by direct or indirect means. In the current study, in which the riboflavin solution leached from the full-thickness stromal tissue into the swelling medium and was continually removed, no such changes in hydrodynamic behaviour were observed between the treated and untreated specimens. Our findings therefore confirm both our x-ray scattering data and our theoretical assumption that collagen fibril surfaces are too widely separated to allow direct interfibrillar linkages to occur (Figure 8D), and indicate that the formation of cross-links between collagen molecules and proteoglycan core proteins (Figure 8E) is unlikely. On the other hand, the absence of any noticeable abnormality in the swelling behaviour of cross-linked corneas allowed to swell freely in saline solution seems inconsistent with the restricted swelling of similarly treated corneas placed in a humidity chamber (where no leaching of proteoglycans occurs) [17]. This could be explained by the existence of indirect cross-linkage between collagen fibrils via their attached proteoglycan core proteins (Figure 8G). Furthermore, comparison of our swelling study with that of Wollensak et al. [20] has for the first time produced scientific evidence that may explain why the beneficial effects of improved corneal thickness and transparency seen in riboflavin/UVA cross-linked bullous keratopathy corneas, fade with time [14], [15], [16]. It seems that the transient decrease in stromal thickness following treatment, may be largely attributable to the deturgescent effect of the dextran, which once removed from the tissue allows the osmotic balance of the cornea to be gradually restored. Indeed bullous keratopathy corneas dehydrated with 40% glucose for 24 hour prior to cross-linking produced much longer-lasting reductions in corneal thickness (up to 8 months) [45] than those cross-linked without prior dehydration [14], [15], presumably due to the corneal oedema limiting the penetration of riboflavin (and hence dextran) into the deeper layers of the cornea during the standard cross-linking procedure.

By a process of elimination, it seems that riboflavin/UVA cross-links most likely occur within (Figure 8A) and between (Figure 8C) collagen molecules positioned at the surface of fibrils, within proteoglycan core proteins (Figure 8F) and between proteoglycan core proteins attached to an individual fibril or adjacent fibrils (Figure 8G). The former (Figure 8A and C) would explain both the increased stiffness of the tissue after riboflavin/UVA cross-linking [7], [8] and its reported [9] and here confirmed resistance to enzymatic attack.

With regard to the latter mentioned scenarios (Figures 8F and G), Spoerl et al [46] have shown that treatment of corneas with α-amylase (to remove interfibrillar substances) results in reduced tissue stiffness, thereby indicating that the interaction between collagen fibrils and the interfibrillar substances plays a role in determining the biomechanical properties of the cornea. Cross-links formed within and between the core proteins of proteoglycans (Figures 8F and G) or other fibrillar and FACIT collagens present in the corneal stroma may therefore be expected to contribute to the observed increase in tissue stiffness following riboflavin/UVA cross-linking.

**Figure 9:**
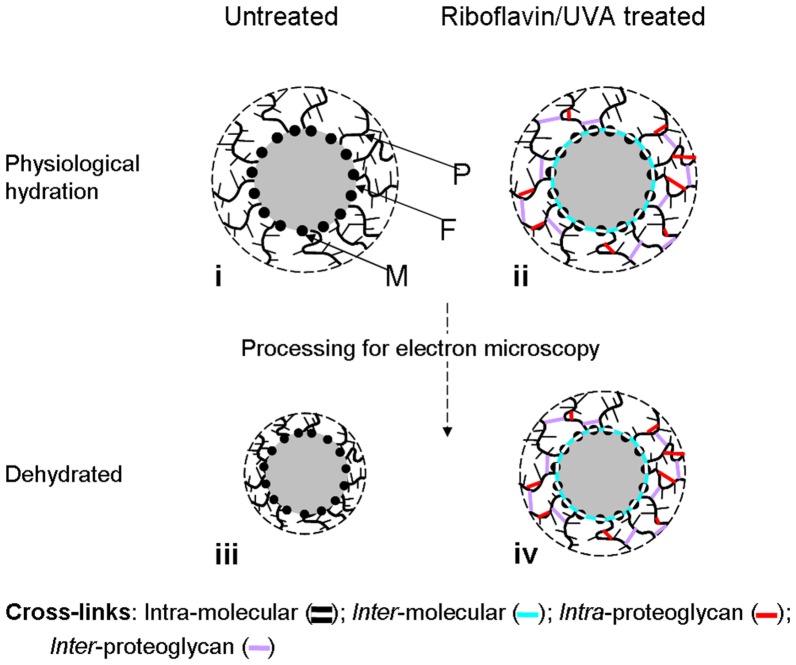
Schematic showing likely collagen shrinkage during electron microscopy processing of cross-linked and non-cross-linked corneas. (i) The theoretical structure of a coated collagen fibril (F) in the corneal stroma (as proposed by Fratzl and Daxer [48]). The coating (outer limit shown as a broken line), consists mainly of proteoglycans (P) which are attached to the fibril and form a porous network with fractal dimension. We propose that riboflavin/UVA induced cross-links are formed within the coating of the collagen fibril between proteoglycan core proteins and/or on the surface of the fibril within and between collagen molecules (M) (ii) and prevent the usual shrinkage associated with tissue dehydration during electron microscopy processing (iii and iv). Hence, when viewed by electron microscopy, collagen fibrils in riboflavin/UVA treated corneas (iv) may misleadingly appear larger in diameter than those in untreated corneas (iii).

The formation of cross-links at the above mentioned sites is further supported by the contradictory evidence of collagen fibril diameter changes following cross-linking when measured here by x-ray scattering or by others using electron microscopy [47]. In our x-ray scattering study, the absence of any detectable change in collagen fibril diameter (at any tissue depth) following treatment was consistent with the fact that no changes in intermolecular spacing were observed. However, electron microscopy studies found relatively larger fibrils in the anterior stroma (and also to a lesser extent the posterior stroma) of riboflavin/UVA treated rabbit corneas compared to their untreated controls [47]. We hypothesise that the discrepancy between these two studies is due to both the location of the riboflavin/UVA induced cross-links and the processing techniques involved in electron microscopy which result in tissue dehydration. As shown by Fratzl and Daxer [48] the drying of the cornea appears to occur in two-stages. At physiological hydration the interfibrillar matrix alone is dehydrated but when the cornea reaches its critical point of drying (H = 1) the fibrils themselves become dehydrated. The change in fibril diameter between H = 1 and H = 0 (dry state) corresponds to the difference in the diameters measured at physiological hydration by x-ray scattering [40] or electron microscopy (using a low temperature resin embedding technique) [49] and those measured by electron microscopy for fixed and stained samples [50]. Based on the fact that fibrils in the hydrated matrix of the normal cornea are never seen to be touching [50], Fratzl and Daxer [48] built on the ideas of Twersky [51] to propose a model (Figure 9i) in which collagen fibrils of a constant diameter are themselves surrounded by an outer coating (consisting predominantly of proteoglycans bound at specific sites along the fibril [52]), the diameter of which is determined by water content. Therefore, the apparent 10% reduction in fibril diameter which occurs following electron microscopy processing [40] may in fact be partially due to shrinkage of this outer coating rather than shrinkage of the fibril itself (Figure 9ii). Using this model and supported by the findings in this paper, we hypothesise that the cross-links formed during riboflavin/UVA therapy occur at the surface of the fibril and within the outer coating of the fibril (Figure 9iii) and thereby restrict its shrinkage during tissue dehydration (Figure 9iv). Such a model would explain the absence of any detectable difference in collagen fibril diameter between treated and untreated rabbit, pig and human corneas when measured at near physiological hydration using x-ray scattering and also the comparably larger diameter fibrils in cross-linked corneas (up to 12% larger in rabbits [47] and 15% larger in hens [43]) compared to untreated corneas when examined in a dehydrated state by electron microscopy.

In this study we have provided the first evidence that riboflavin/UVA induced cross-links do not exist between or within collagen fibrils but possibly occur at the surface of the fibrils and within the proteoglycan rich coating surrounding them. The results of this study lead us to believe that the longevity of improved corneal stability following cross-linking is dependant on the rate of collagen turnover in the cornea; this is believed to be an extremely slow process, similar to that of skin and cartilage collagen (whereby the half life is estimated to be 15 and 117 years respectively) [53] and is likely to be even slower in cross-linked corneas as a result of the increased resistance to enzymatic digestion.
